# Shear-wave elastography: a new potential method to diagnose ulnar neuropathy at the elbow

**DOI:** 10.1007/s00330-018-5517-9

**Published:** 2018-06-01

**Authors:** Łukasz Paluch, Bartłomiej Noszczyk, Żaneta Nitek, Jerzy Walecki, Katarzyna Osiak, Piotr Pietruski

**Affiliations:** 1 Department of Radiology, Medical Centre of Postgraduate Education, Gruca Orthopaedic and Trauma Teaching Hospital, Otwock, Poland; 2 Department of Plastic and Reconstructive Surgery, Medical Centre of Postgraduate Education, Prof. W. Orlowski Memorial Hospital, Czerniakowska 231 Street, 00-416 Warsaw, Mazowieckie Poland; 3 Department of Applied Pharmacy and Bioengineering, Medical University of Warsaw, Warsaw, Poland

**Keywords:** Elastography, Cubital tunnel syndrome, Ulnar nerve, Ulnar nerve entrapment syndrome, Ulnar neuropathies

## Abstract

**Objectives:**

The primary aim of this study was to verify if shear-wave elastography (SWE) can be used to diagnose ulnar neuropathy at the elbow (UNE). The secondary objective was to compare the cross-sectional areas (CSA) of the ulnar nerve in the cubital tunnel and to determine a cut-off value for this parameter accurately identifying persons with UNE.

**Methods:**

The study included 34 patients with UNE (mean age, 59.35 years) and 38 healthy controls (mean age, 57.42 years). Each participant was subjected to SWE of the ulnar nerve at three levels: in the cubital tunnel (CT) and at the distal arm (DA) and mid-arm (MA). The CSA of the ulnar nerve in the cubital tunnel was estimated by means of ultrasonographic imaging.

**Results:**

Patients with UNE presented with significantly greater ulnar nerve stiffness in the cubital tunnel than the controls (mean, 96.38 kPa vs. 33.08 kPa, *p* < 0.001). Ulnar nerve stiffness of 61 kPa, CT to DA stiffness ratio equal 1.68, and CT to MA stiffness ratio of 1.75 provided 100% specificity, sensitivity, positive and negative predictive value in the detection of UNE. Mean CSA of the ulnar nerve in the cubital tunnel turned out to be significantly larger in patients with UNE than in healthy controls (*p* < 0.001). A weak positive correlation was found in the UNE group between the ulnar nerve CSA and stiffness (R = 0.31, *p* = 0.008).

**Conclusions:**

SWE seems to be a promising, reliable and simple quantitative adjunct test to support the diagnosis of UNE.

**Key Points:**

• *SWE enables reliable detection of cubital tunnel syndrome*

• *Significant increase of entrapped ulnar nerve stiffness is observed in UNE*

• *SWE is a perspective screening tool for early detection of compressive neuropathies*

## Introduction

Cubital tunnel syndrome, also referred to as ulnar neuropathy at the elbow (UNE), is the second most common peripheral entrapment neuropathy after carpal tunnel syndrome. It can be defined as a compression of the ulnar nerve at the level of the elbow or in its direct proximity [1–4]. The ulnar nerve may be compressed at the cubital tunnel inlet by the medial intermuscular septum and an aponeurotic band referred to as the arcade of Struthers. Moreover, the nerve may be entrapped in the tunnel by the Osborne's band, a retinaculum that extends from the medial epicondyle to the olecranon, and by the aponeurosis of the flexor carpi ulnaris (FCU); this is postulated to be the most common site of the entrapment [5–7]. Finally, the ulnar nerve may be compressed distally, at the tunnel outlet, by the two heads of the FCU muscle [7].

Idiopathic neuropathy seems to be the most common cause of UNE among many potential aetiological factors of this condition [7, 8]. Ulnar neuropathy may also result from habitual elbow flexion, acute or repetitive trauma, excessive strain, presence of mass-like lesions, degenerative arthritis and snapping triceps syndrome. The list of intrinsic aetiological factors includes diseases of the thyroid, diabetes mellitus, alcohol abuse, rheumatoid arthritis and other systemic inflammatory diseases, to mention a few [5, 7, 9–11].

Because of its high incidence in the general population and a broad spectrum of symptoms that may interfere with patients’ ability to work and their activities of daily living, UNE constitutes a significant clinical problem [7, 8, 12]. A number of healthcare professionals with various specialities, among them general practitioners, plastic surgeons, orthopaedic surgeons and neurologists, are involved in the evaluation of UNE. Although no consensus regarding a management algorithm for UNE has been reached thus far, a recent meta-analysis conducted by Mowlavi et al. demonstrated that clinical work-up is crucial to establish the diagnosis, to determine the severity of ulnar neuropathy and to choose an appropriate treatment strategy [12]. The diagnosis is based primarily on detailed analysis of medical history and physical examination. According to many authors, electrodiagnostic studies constitute an essential component of the evaluation, as they can be used to confirm the diagnosis and to assess the severity of nerve damage. Additional tests, such as ultrasonography (US), plain film radiography and magnetic resonance imaging (MRI), are recommended in patients with vague clinical presentation or during preoperative work-up [1, 5–7, 13–25]. Based on published evidence we can expect that also diffusion tensor imaging may be soon added to the peripheral nerve assessment armamentarium [26–31].

During recent years, sonoelastography, a relatively new imaging technology to quantify tissue stiffness, has been gaining a growing interest of researchers and healthcare professionals. Since its introduction by Ophir and colleagues in 1991, sonoelastography has evolved considerably and found application in many clinical disciplines; other potential uses of sonoelastography are still a subject of ongoing research [32, 33]. One highly promising application of sonoelastography, in particular shear-wave elastography (SWE), is the evaluation of peripheral nerve elasticity in patients with entrapment syndromes. Several authors reported the use of elastography in the diagnosis of median, sciatic and tibial compressive neuropathies [34–43]. However, to the best of our knowledge, potential application of this technique as a method to detect UNE has not been studied thus far.

The aim of this study was to use SWE for the quantification of ulnar nerve elasticity in patients with UNE and in healthy controls, and to verify if this technique may find application in clinical practice as an instrument to detect compressive neuropathy.

## Materials and methods

### Study protocol

Protocol of the study was approved by the local bioethics committee, and written informed consent was sought from all participants. A plastic surgeon assigned the study subjects to one of two groups, based on the inclusion criteria presented in Table 1.

**Table 1:** Inclusion criteria of the study

UNE group	Control group
UNE symptoms present on physical examination	No evidence of UNE symptoms on physical examination
UNE confirmed by EDX	UNE excluded by EDX
No history of elbow surgery No history of humeral or ulnar fracture No systemic neurological disorder No thyroid disorder No diabetes mellitus No current pregnancy	

*EDX*, Electrodiagnostic testing; *UNE*, Ulnar neuropathy at the elbow

Each study participant was subjected to US and SWE, both conducted with a Toshiba iAplio 900 ultrasonograph equipped with a 5-18 MHz transducer (Canon Medical Systems). Both US and SWE were carried out during the same visit, by the same radiologist with more than 4 years of experience in both diagnostic methods, blinded to subjects’ clinical history, physical and results of electrodiagnostic testing (EDX). The examination was performed in a seated position, with the subject facing the examiner. The examined arm was kept downwards alongside the trunk, with flexed elbow and forearm resting on the knee in a supine position. To control and minimise the pressure applied onto skin surface, an even layer of hydrogel was applied onto the examined area.

During B-mode US examination of the cubital tunnel, the transducer was maintained perpendicularly to the ulnar nerve. Transverse cross-sections of the ulnar nerve were acquired at medial epicondyle level to determine the cross-sectional area of the nerve (CSA, in mm^2^). To measure the CSA, the ulnar nerve margin was traced along the inner border of perineural echogenic rim, corresponding to the perineurium around the hypoechoic nerve.

**Fig. 1:**
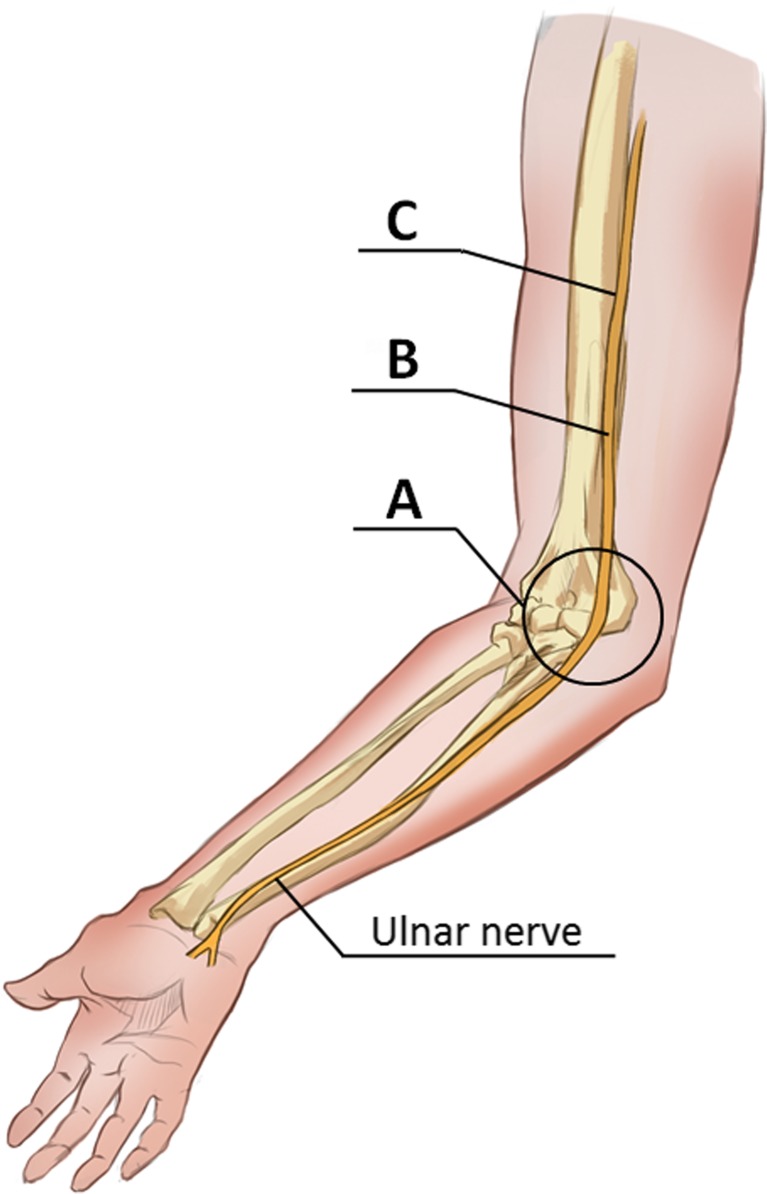
Ulnar nerve segments subjected to elastographic examination. A, Cubital tunnel at medial epicondyle level; B, Posteromedial arm, 5 cm proximally to the cubital tunnel inlet (distal arm level); C, Posteromedial arm, 10 cm proximally to the cubital tunnel inlet (mid-arm level)

**Fig. 2:**
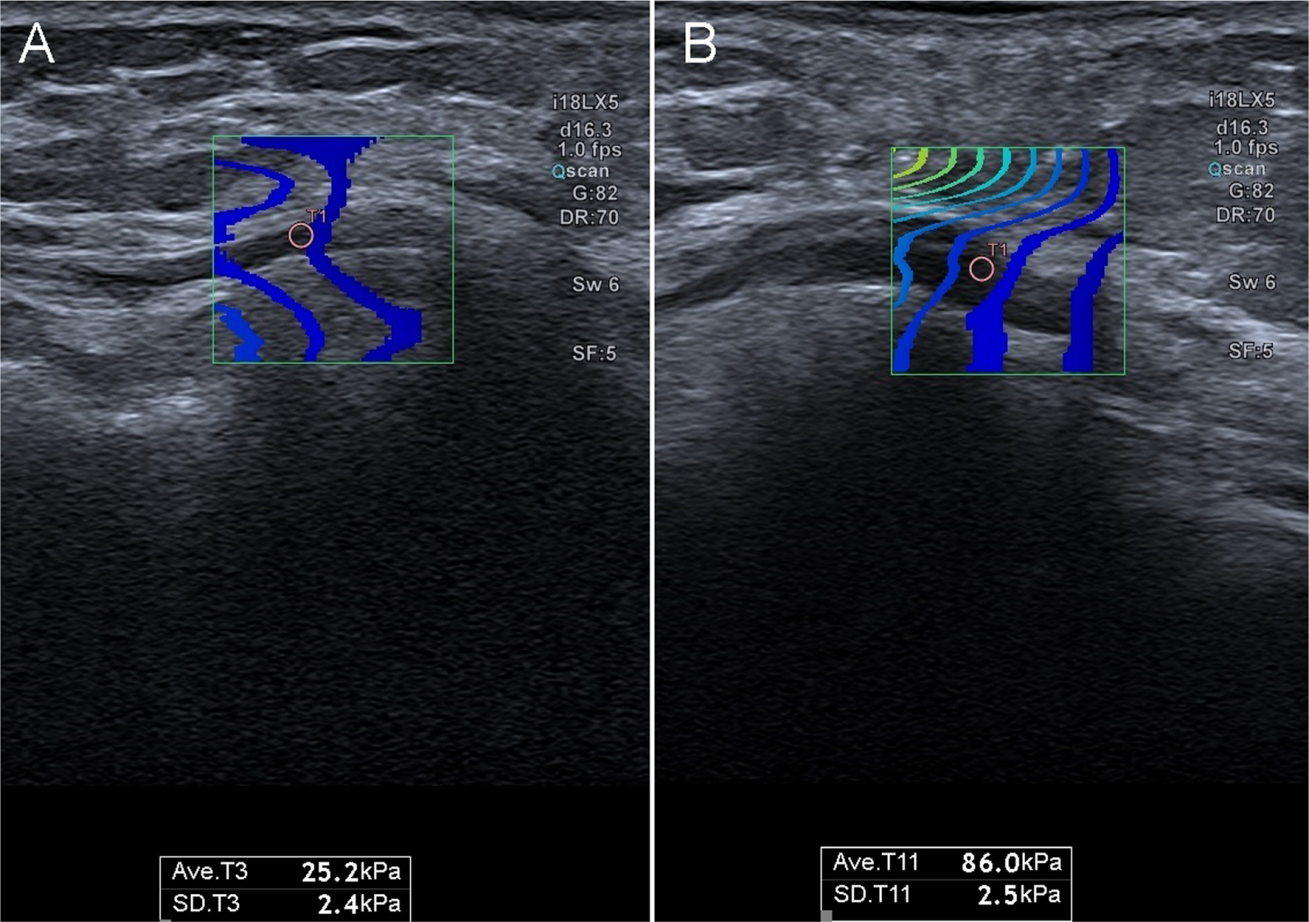
Elastographic presentation of ulnar nerve at the elbow level in (**A**) healthy individual (low SWE value), and (**B**) in patient with compressive neuropathy (high SWE value)

Ulnar nerve stiffness was measured at three levels labelled with a skin marker prior to the examination: in the cubital tunnel at the level of medial epicondyle and at a posteromedial aspect of the arm, 5 cm and 10 cm proximally to the cubital tunnel inlet (i.e. on the distal and mid-arm, respectively) (Fig. 1). The course of the ulnar nerve at each level was localised on B-mode scans. Then, the transducer was rotated 90 degrees to obtain SWE-mode images of the nerve in a longitudinal axis. The transducer was always kept parallel to skin surface, and special attention was paid to minimise the pressure and to avoid a disruption of the hydrogel layer. Nerve stiffness at each level was measured three times with 2-3 min intervals and mean measurement was used for further processing; during the examination, the ulnar nerve was always positioned in the centre of the 1x1 cm Q-box, set in such way that no underlying bone surfaces were captured on longitudinal ultrasonographic scans. Shear modulus data for the selected circular region of interest (ROI, 2 mm in diameter) were acquired automatically by the ultrasonographic software; the results were expressed in kilopascals (kPa). Representative elastographic images are presented in Fig. 2.

### Statistical analysis

Normal distribution of the study variables was verified with Shapiro-Wilk test. Statistical characteristics of continuous variables were presented as means, medians and ranges. The characteristics of qualitative variables were shown as numbers and percentages. The significance of intergroup differences in the statistical characteristics of continuous variables was verified with Mann-Whitney U-test and Kruskal-Wallis test with Dunn post-hoc tests, whereas the significant of intragroup differences was tested with Wilcoxon signed-rank test. Diagnostic accuracy of ulnar nerve stiffness and CSA in the detection of UNE was verified by means of receiver operating characteristic (ROC) analysis. Sensitivity, specificity, positive and negative predictive value (PPV and NPV, respectively) of each potential predictor of UNE were calculated, along with the area under the ROC curve (AUC) and its 95% confidence interval (95% CI). All calculations were carried out with Statistica 10 package (StatSoft), with the threshold of statistical significance set at *p* ≤ 0.05.

## Results

UNE group included 34 patients with mean age of 59.35 years (range, 36-85 years), among them six men (mean age, 62 years; range, 48-85 years) and 28 women (mean age, 58,79 years; range, 36-80 years), with confirmed ulnar compressive neuropathy at the elbow level. Control group comprised 38 healthy volunteers with mean age of 57.42 years (range, 38-84 years), among them three men (mean age, 53.67 years; range, 38-75 years) and 35 women (mean age, 57.74 years; range, 38-84 years), in whom UNE was excluded based on the results of physical examination and EDX.

**Fig. 3:**
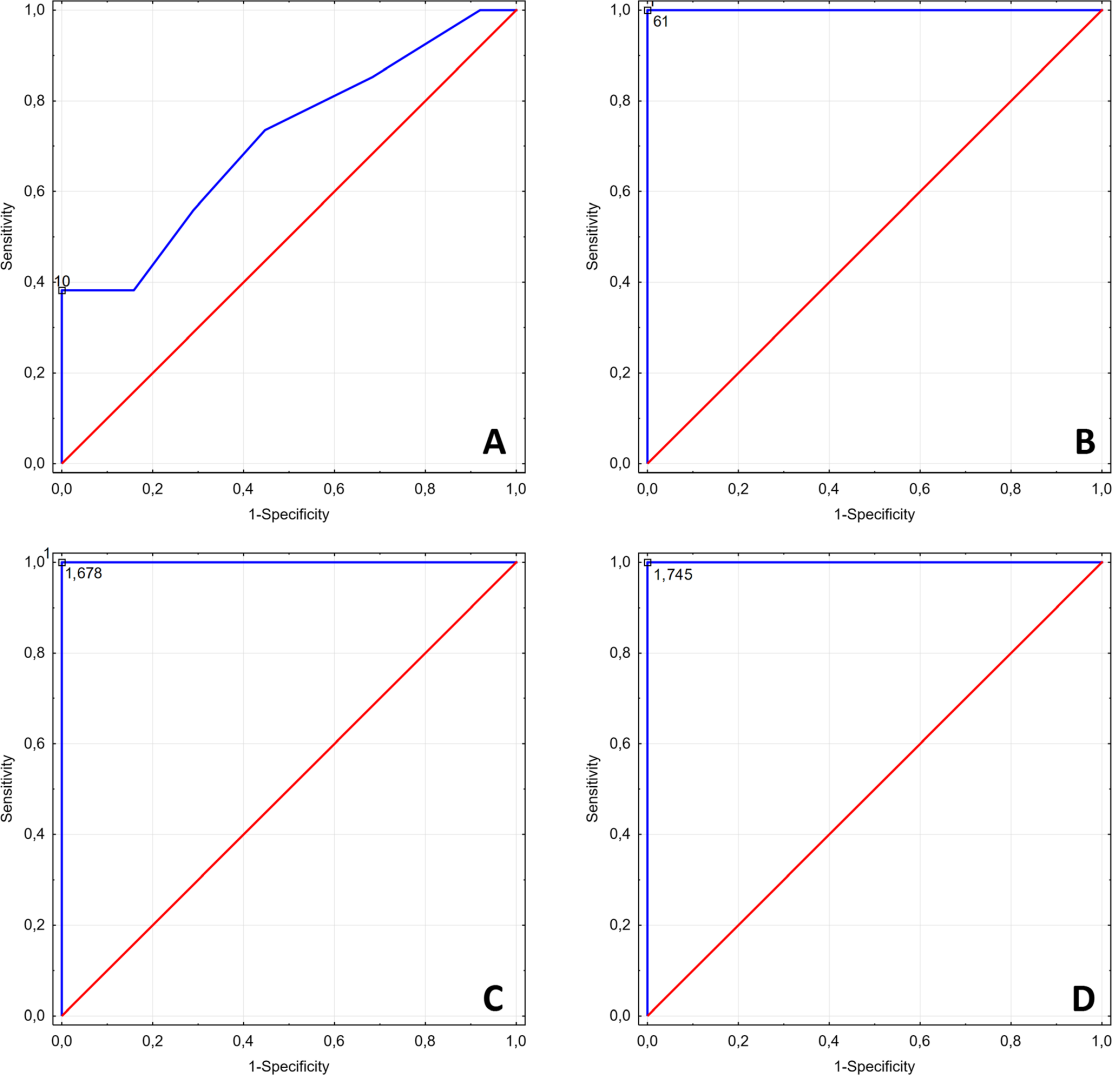
ROC curve illustrating diagnostic accuracy of (**A**) the cross-sectional area of ulnar nerve (10 mm^2^), (**B**) ulnar nerve stiffness at the entrapment site (61 kPa), (**C**) CT to DA ratio (1.68), and (**D**) CT to MA ratio (1.75) in the detection of UNE

The CSA of the ulnar nerve at the cubital tunnel level turned out to be significantly larger in patients with UNE than in the controls (mean, 9.03 mm^2^; median, 8 mm^2^; range, 5-18 mm^2^ vs. mean, 6.47 mm^2^; median, 6 mm^2^; range, 3-9 mm^2^; *p* < 0.001). The cut-off value for the CSA of the ulnar nerve at the cubital tunnel level, which most accurately distinguished between the individuals with the entrapment neuropathy and without was 10 mm^2^; ROC analysis demonstrated that this value provided 38.2% sensitivity, 100% specificity, 100% PPV and 64.4% NPV in the detection of UNE (Fig. 3).

The results of SWE measurements are summarised in Table 2. In any patient the differences between measurements that were repeated at each level with 3 minutes intervals did not exceed 2 kPa (SD ≥ 1.15). Compared to the controls, patients with UNE presented with significantly greater ulnar nerve stiffness at each examined level. In the UNE group, mean ulnar nerve stiffness at the entrapment site in the cubital tunnel (96.38 kPa) was significantly greater than at the distal arm and mid-arm (38.79 kPa and 39.06 kPa, respectively; *p* < 0.001). In the healthy controls, no statistically significant differences were found in ulnar nerve stiffness at various levels (*p* = 0.779).

**Table 2:** Elastographic estimates of ulnar nerve stiffness (in kPa) in healthy volunteers (control group) and patients with confirmed cubital tunnel syndrome (UNE group)

Ulnar nerve SWE value	Control group (n=38)				UNE group (n=34)				*p*
	Mean	Median	Range	SD	Mean	Median	Range	SD	
Cubital tunnel level	33.08	31	19-51	10.13	96.38	98	61-121	9.62	<0.001*
Distal arm level	33.32	33	21-51	9.13	38.79	36,5	21-65	11.33	0.041
Mid-arm level	33.18	31	19-52	8.82	39.06	36	20-61	10.45	0.009

*Statistically significant intergroup difference

Aside from the comparison of crude SWE parameters for the entrapment site, we also analysed their normalised values, obtained by dividing ulnar nerve stiffness at the cubital tunnel level (CT) by its stiffness at the distal arm (CT to DA ratio) or mid-arm (CT to MA ratio). The values of both ratios turned out to be significantly higher in patients with UNE than in the controls (*p* < 0.001) (Table 3).

**Table 3:** Normalised values of ulnar nerve stiffness (ratios) in healthy volunteers (control group) and patients with confirmed cubital tunnel syndrome (UNE group)

Ulnar nerve SWE ratio	Control group (n=38)		UNE group (n=34)		*p*
	Median	Range	Median	Range	
Cubital tunnel level : Distal arm level	1.0	0.8-1.2	2.8	1.7-4.0	<0.001*
Cubital tunnel level : Mid-arm level	1.0	0.7-1.2	2.7	1.7-4.1	<0.001*

*Statistically significant intergroup difference

ROC curves illustrating the diagnostic accuracy of elastographically determined ulnar nerve stiffness at the cubital tunnel level, CT to DA and CT to MA ratios in the detection of UNE are presented in Fig. 3. Ulnar nerve stiffness at the cubital tunnel level equal 61 kPa provided 100% specificity, sensitivity, PPV and NPV in the detection of UNE. Equally excellent indices of diagnostic accuracy were also obtained for the CT to DA ratio of 1.68, and CT to MA ratio of 1.75 (Fig. 3).

A weak positive correlation was found in the UNE group between the CSA of the ulnar nerve and its stiffness at the site of compression (R = 0.31, *p* = 0.008). Moreover, the CSA of the ulnar nerve correlated significantly with the values of CT to DA (R = 0.31, *p* = 0.007) and CT to MA ratios (R = 0.30, *p* = 0.010).

## Discussion

We hypothesised that SWE can be used for an objective evaluation of oedema and fibrosis associated with ulnar nerve compression. The study showed clearly that patients with UNE presented with significantly greater ulnar nerve stiffness at the cubital tunnel level than healthy controls (mean, 96.38 kPa vs. 33.08 kPa). Furthermore, stiffness of the ulnar nerve in UNE patients turned out to be significantly greater at the entrapment site than at the mid- and distal arm; however, a similar relationship was not observed in the controls. ROC analysis demonstrated that UNE could be accurately diagnosed whenever stiffness of the ulnar nerve at the entrapment site equalled 61 kPa or more. However, recall that ulnar nerve stiffness may vary from patient to patient due to the modulatory effect of additional factors, such as sex, age, BMI, occupation, and upper limb physiognomy. Thus, to boost the diagnostic accuracy of SWE, instead of using the crude value of ulnar nerve stiffness at the entrapment site, we recommend a normalised value of this parameter; dividing ulnar nerve stiffness at the entrapment site by the stiffness of its intact segment, one can eliminate individual variability in the elastographic parameters. In this study, we divided ulnar nerve stiffness at the entrapment site by its stiffness at the distal and mid-arm, obtaining CT to DA and CT to MA ratios, respectively. Similar to the crude value of ulnar nerve stiffness, also the values of both ratios were significantly higher in patients with CTS than in the controls. Moreover, ROC analysis demonstrated that the cut-off values for CT to DA and CT to MA ratios (1.68 and 1.75, respectively) accurately distinguished between patients with compressive ulnar neuropathy and without.

As mentioned before, the diagnosis of UNE is based primarily on medical history and physical examination. Although this diagnosis is usually confirmed by EDX, US due to recent implementation of high-frequency transducers enabling high-resolution images of peripheral nerves is emerging as a complementary method to evaluate patients with compressive neuropathies, e.g. UNE [5, 7, 15, 17, 22–25, 44, 45]. The hallmark sonographic features of entrapment neuropathy include enlargement of the nerve, its hypoechoic swelling proximal to the compression site, loss of normal fascicular pattern and, not infrequently, oedema of adjacent soft tissues [7, 13, 20, 22, 23]. US is also suitable for the detection of space-occupying masses. Importantly, during the examination, ultrasonographic images of the affected limb can be compared with those of the contralateral extremity. High-resolution US was shown to be more cost- and time-effective than MRI, and its availability is markedly higher. Noticeably, US can be used for both static and dynamic assessment of the ulnar nerve; this may be particularly helpful in patients with nerve subluxation or snapping triceps syndrome [5, 7]. Previous studies demonstrated that ultrasonographically determined ulnar nerve CSA was a feasible indicator for UNE [5, 25, 46]; ulnar nerve CSA at the elbow level equal to 8.3 mm^2^ provided high specificity and sensitivity in the detection of ulnar nerve entrapment [5, 17, 24, 25]. Our findings seem to be consistent with these observations. Not only did the mean CSA of the ulnar nerve at the cubital tunnel level turn out to be significantly larger in patients with UNE than in healthy controls (9.03, range, 5-18 mm^2^ vs. 6.47, range, 3-9 mm^2^), but also a significant positive correlation was found between this parameter and the elastographically determined ulnar nerve stiffness at the entrapment site. The latter observation seems to confirm the applicability of SWE to UNE diagnosis. The principal drawback of peripheral nerve ultrasonography is the lack of a standardised examination protocol (examination technique, type of transducer, wave frequency) [7]. Moreover, the examination is to a large degree operator-dependent, and thus, its result can be considered reliable only if conducted by an experienced examiner.

Our findings are consistent with the results of other studies that analysed potential application of elastography in the evaluation of compression neuropathies; also, those studies demonstrated that compression may contribute to greater stiffness of the entrapped nerve [34–43]. In our opinion, SWE constitutes a valuable supplement to ultrasonographic examination of peripheral nerves, also in patients with UNE [14]. Elastography is both a time- and cost-effective method with a relatively steep learning curve. Elastographic findings add considerably to the results of US, providing quantitative data on the nerve stiffness during both static and dynamic examination. In addition, it can determine the level of nerve entrapment. In the future, with its growing availability in routine clinical practice, SWE may be used for the monitoring of treatment outcomes in UNE patients. The numerous advantages of SWE make it a perspective screening tool for early detection of compressive neuropathies, with potential application in occupational medicine. Prompt introduction of adequate preventive measures in persons at risk or implementation of conservative treatment in individuals with early stages of the neuropathy may prevent its long-term consequences, such as chronic disability.

The main limitation of this study may stem from the fact that ulnar nerve stiffness was determined by one radiologist, and thus, we were unable to estimate inter-observer reliability of the results. During the course of another study, which is soon to be conducted at our centre, we will analyse both inter- and intra-observer reliability of SWE measurements taken at various levels of ulnar and median nerves. Interestingly, patients from UNE group presented with significantly greater ulnar nerve stiffness proximally to the compression site than the controls. Because of the preliminary characteristic of our study and lack of evidence from similar study, we are unable to provide an unequivocal explanation for this phenomenon. The exact pathophysiology of compressive neuropathy is still not fully understood. It is commonly accepted that increased pressure on a peripheral nerve affects its microcirculation and, therefore, blood supply. Resultant epineural ischaemia contributes to impaired venous outflow and stasis. Chronic compression may predispose to capillary leakage and neural oedema, which further aggravates the harmful effects of the primary causal factor. With time, this mechanism induces inflammation, fibrosis, demyelination and eventually, after a remyelination failure, axonal integrity loss [7, 47–49]. Perhaps, this process involves the segment located proximally to the compression site to a larger degree than previously supposed, which manifests as greater stiffness of the nerve, likewise in our series. This would suggest that elastography may be a highly sensitive test to detect structural anomalies in peripheral nerves. This issue definitively needs to be addressed in future studies.

The results of this preliminary study justify further research on the application of SWE as a diagnostic instrument in ulnar neuropathies. The future studies should involve larger groups of patients and verify whether SWE is suitable for the staging of ulnar nerve dysfunction and if the elastographic indices correlate with the routinely used EDX-based scale of UNE severity. Moreover, future research should corroborate if elastography could be used as a screening tool for peripheral neuropathies and whether it may find application as a method to monitor the treatment outcomes.

## Conclusions

To summarise, this study demonstrated for the first time that shear wave elastography can be used to diagnose cubital tunnel syndrome. Patients with entrapment syndrome presented with significantly greater ulnar nerve stiffness at the elbow level than healthy controls. These findings imply that SWE has a potential to become a routine diagnostic instrument to detect ulnar neuropathy in a clinical setting.

## Abbreviations

CSA: Cross-sectional areas
CT: Cubital tunnel
DA: Distal arm
EMG: Electromyography
FCU: Flexor carpi ulnaris
MA: Mid-arm
MRI: Magnetic resonance imaging
ROI: Region of interest
SWE: Shear-wave elastography
UNE: Ulnar neuropathy at the elbow
US: Ultrasonography
